# Generic estimator of biomass concentration for *Escherichia coli* and *Saccharomyces cerevisiae* fed-batch cultures based on cumulative oxygen consumption rate

**DOI:** 10.1186/s12934-019-1241-7

**Published:** 2019-11-05

**Authors:** Renaldas Urniezius, Arnas Survyla, Dziugas Paulauskas, Vladas Algirdas Bumelis, Vytautas Galvanauskas

**Affiliations:** 1 Department of Automation, Kaunas University of Technology, 51367 Kaunas, Lithuania; 2 Biopharmaceutical Division of Centre for Innovative Medicine, 08406 Vilnius, Lithuania

**Keywords:** Biomass estimator, Stoichiometry, Relative entropy, Microbial cultivation

## Abstract

**Background:**

The focus of this study is online estimation of biomass concentration in fed-batch cultures. It describes a bioengineering software solution, which is explored for *Escherichia coli* and *Saccharomyces cerevisiae* fed-batch cultures. The experimental investigation of both cultures presents experimental validation results since the start of the bioprocess, i.e. since the injection of inoculant solution into bioreactor. In total, four strains were analyzed, and 21 experiments were performed under varying bioprocess conditions, out of which 7 experiments were carried out with dosed substrate feeding. Development of the microorganisms’ culture invariant generic estimator of biomass concentration was the main goal of this research.

**Results:**

The results show that stoichiometric parameters provide acceptable knowledge on the state of biomass concentrations during the whole cultivation process, including the exponential growth phase of both *E. coli* and *S. cerevisiae* cultures. The cell culture stoichiometric parameters are estimated by a procedure based on the Luedeking/Piret-model and maximization of entropy. The main input signal of the approach is cumulative oxygen uptake rate at fed-batch cultivation processes. The developed noninvasive biomass estimation procedure was intentionally made to not depend on the selection of corresponding bioprocess/bioreactor parameters.

**Conclusions:**

The precision errors, since the bioprocess start, when inoculant was injected to a bioreactor, confirmed that the approach is relevant for online biomass state estimation. This included the lag and exponential growth phases for both *E. coli* and *S. cerevisiae*. The suggested estimation procedure is identical for both cultures. This approach improves the precision achieved by other authors without compromising the simplicity of the implementation. Moreover, the suggested approach is a candidate method to be the microorganisms’ culture invariant approach. It does not depend on any numeric initial optimization conditions, it does not require any of bioreactor parameters. No numeric stability issues of convergence occurred during multiple performance tests. All this makes this approach a potential candidate for industrial tasks with adaptive feeding control or automatic inoculations when substrate feeding profile and bioreactor parameters are not provided.

## Background

Biotechnology industry development over the last years made quality assurance more stringent for pharmacy production [1]. As a tool to resolve process data distortion and prevent operator from accidently making mistakes, bioengineering solutions help to automate tasks, which results in rise of cultivation process performance and quality. To strengthen product quality, to more efficiently acquire coefficient values, to improve safety and flexibility of adaptive feedback control, the soft/noninvasive sensors [2] become a rational choice for development of sustainable engineering solutions. Implementation of feedback control system requires a feedback signal from soft sensors or estimators that provide parameters [3], which are unavailable to be directly measured online [4]. The control algorithm and the feedback signal consider the product and the main characteristics of bioprocess parameters—the biomass concentration and the specific growth rate [5, 6].

This study delves into biomass estimator development based on stoichiometric parameters and Luedeking–Piret model. The cell’s yields and stoichiometry both form a generic information, which is an acceptable candidate to be included in estimators when the microorganisms culture does not change from experiment to experiment. Depending on stoichiometry, the estimator of biomass concentration can be used to automatically inject the inoculant solution at a predefined level of the optical density in bioreactor medium. At this point, cumulative oxygen uptake rate signal from an off-gas analyzer is informative to determine the biomass concentration.

The biomass estimator described in this study includes optimization algorithm, which returns the stoichiometric parameters of the controlled culture. The algorithm refers to several optimization criteria and is based on a gray box model originating from Luedeking–Piret model. Then offline maximization of entropy leads to satisfactory parameters values for estimation procedure, which is then applied to *Escherichia coli* bacteria and *S. cerevisiae* yeast cultures. In other words, the stoichiometry optimization algorithm must be performed once for each strain to determine the necessary coefficients. These coefficients can be later used in the subsequent experiments to estimate biomass concentration online, unless the strain does not change. Such offline analysis can be considered as an estimator tuning algorithm for a specific microorganisms’ culture.

The “Materials and methods” section describes the materials, strains and the bioreactor system operating conditions. The “Comparative analysis of biomass estimators” section reviews literature references of the off-gas analysis approaches and introduces the motivation for this study. The “General mathematical model of stoichiometric parameters estimation” section layouts the derivation of the bioengineering approach for both the offline (stoichiometry) analysis and the online (biomass concentration) analysis stages. It also resolves a general formulation of the oxygen consumption for biomass maintenance coefficient, which is relevant for both *E. coli* and *S. cerevisiae* cultures. The “Experimental validation” section provides experimental proof of the developed stoichiometry coefficients offline identification and the biomass concentration online estimation algorithms. The “Conclusions” section discusses the results and concludes the final statements of this study.

## Materials and methods

### Cell strain’s

Four types of strain cultivation were analyzed in this work to verify biomass estimation. *S. cerevisiae* (no DY7221) strain was used as representative of yeasts cells. The recombinant strains *E. coli* BL21(DE3) pET9a-IdeS, *E. coli* BL21 (DE3) pET21-IFN-alfa-5 (cloning of fused gene into bacterial systems with strong bacterio-phage T7 promoter, pET21a + plasmid) [7] and *E.coli* BL21(DE3) pLysS [8] were used in bacterial cultivations.

### Medium and culture conditions

In order to check biomass estimator’s reliability and accuracy, data were collected from different cell strains which have been cultivated in multiple different R&D laboratories, including the laboratory of bioprocessing modeling and management in Kaunas University of Technology. *Saccharomyces cerevisiae* (no DY7221) strain was cultivated in the standard nutrient medium (YPD) [9, 10], which contained 1% yeast extract, 2% Bacto peptone, and 0.1% glucose. The feed solution contained 600 g/kg glucose which increased the solution density to 1.21 g/l.

The medium temperature was maintained at 30 °C and it was monitored by using temperature sensor “Pt100”, and pH was kept constant at 4.9 by addition of NaOH(aq) [11]. Dissolved oxygen tension DOT in the bioreactor was measured by oxygen electrode Mettler Toledo and controlled by shifting stirrer speed from 230 to 600 rpm. The DOT set point was chosen as 30% of air saturation. The air flow was kept around 4 l/min and measured by a mass air flow sensor. The off-gas from bioreactor was measured online by BlueSens gas analyzer (BCpreFerm, BlueSens, Herten, Germany), which has O_2_, CO_2_ and pressure sensors. The culture broth mass was measured online with balanced reactor vessel which contained load cell weight sensor. The initial substrate concentration in the bioreactor was equal to zero, S = 0 g/kg. Hence, after inoculation the substrate solution feeding was started. The cultivation process was performed in 5 l bioreactor.

The cell strain of *E. coli* BL21 (DE3) pET21-IFN-alfa-5 was cultivated in 7 l bioreactor. Cultivation medium was based on minimal mineral medium, which was made of 46.55 g potassium dihydrogen phosphate, 14 g ammonium phosphate dibasic, 5.6 g citric acid monohydrate, 3 ml of concentrate antifoam, 35 g magnesium sulphate heptahydrate, 105 g D (+) glucose monohydrate. The initial volume of medium was 3.7 kg. At the cultivation process the environment parameters were kept constant. The temperature setpoint was 37 °C, DOT set at 20% of air saturation and pH kept at pH 6.8 by addition of NaOH(aq). The stirrer rpm range was from 800 to 1200 rpm, the air flow rage was from 1.75 to 3.75 l/min. In order to increase oxygen transfer rate during cultivation process, pure oxygen flow was provided to bioreactor at range from 0 to 7.5 l/min. The off-gas from bioreactor was measured online by BlueSens.

The other cell strain of *E. coli* BL21 (DE3) pET9a-IdeS was cultivated in 15 l bioreactor. Cultivation medium based as minimal mineral medium. At the cultivation process the environment parameters: temperature set point was 37 °C, DOT set at 30% of air saturation and pH kept at pH 6.98 by addition of NaOH(aq). The stirrer rpm range was from 300 to 750 rpm, the air flow range was from 0.3 to 15 l/min. During the cultivation process pure oxygen flow was provided to bioreactor at range from 0 to 7.5 l/min. The off-gas from bioreactor was measured online by BlueSens.

For diversity of validation, the fourth cell strain was *E. coli* (BL21(DE3) pLysS) [8]. The cultivation medium used as minimal mineral medium composed with (NH_4_)_2_SO_4_, 2.46 g/l; NH_4_Cl, 0.5 g/l; NaH_2_PO_4_ × H2O, 3.6 g/l; Na_2_SO_4_, 2 g/l; K_2_HPO_4_, 14.6 g/l; (NH_4_)_2_-citrate, 1 g/l; 1 M MgSO_4_ solution, 5 ml/l; trace elements solution, 2 ml/l; and no glucose. Initial masses of all cultures were 5 kg. The glucose solution and initial substrate concentration at the bioreactor used same as at cultivation with yeasts, pH kept constant at pH 7 and temperature was regulated to 30 °C. Dissolved oxygen tension DOT was measured by an amperometric oxygen electrode (Mettler–Toledo) and the DOT set point was 30% of the saturation. The size of bioreactor was 15 l working volume (Biostat C, Sartorius Stedim Biotech) and the stirrer speed varied from 100 to 1400 rpm.

## Comparative analysis of biomass estimators

In order to adaptively control and monitor chemical or biotechnological process, it is mandatory to implement a data collection system that provides desired variables at real time with acceptable precision and performance. This requires corresponding equipment, which may be unaffordable, not implementable in system or the required instrument doesn’t exist. Hence, the better alternative is to use soft or noninvasive sensors, which collect measurable variables and estimate unmeasurable parameters [2, 12]. Especially in biotechnology processes, there are complex relationships between process and variables, so the best way to infer online unmeasurable parameters is to use corresponding estimators [4].

Over time, the studies of both bioprocesses and industrial production perspectives have shown that a biomass estimator requires data, which is closely related to biomass growth rate and biomass concentration. It can be indirectly measured online, with well-established and validated devices and soft sensors [4, 13], which are still in development. Oxygen uptake rate (OUR) and carbon dioxide production rate (CPR) are directly related to biomass growth rate and biomass concentration [14, 15]. Oxygen uptake rate (OUR) and carbon dioxide production rate (CPR) data for estimator must be computed from online signals that are reliable and measured directly in bioreactor system. These signals are the concentration of O_2_ and CO_2_ in the off-gas [16]. The proposed noninvasive biomass concentration estimation procedure was intentionally made to not depend on the selection of bioprocess/bioreactor parameters. The approach is valid for aerobic cultures as long as it is possible to obtain the off-gas measurements of sufficient quality.

The main model, dedicated to biomass concentration estimation in this work, is a Luedeking–Piret model derived from the stoichiometric equations for oxygen consumption. It represents relationship between biomass *X* growth/maintenance and oxygen uptake rate in bioreactor [14, 15]:

1 $$OUR\left( t \right) = \alpha \cdot X'\left( t \right) + \beta \cdot X\left( t \right)$$

Stoichiometric coefficients *α* and *β* represent cell’s metabolisms of oxygen consumption and correspond to the yield coefficients of these biochemical conversions. In Eq. (1) coefficient *α* means specific cell’s oxygen consumption yield ($\alpha \equiv Y_{{{\text{o}}_{2} /{\text{X}}}}$) for growth and *β* is a model parameter termed as oxygen consumption for maintenance ($\beta \equiv {\text{m}}_{{{\text{o}}_{2} /{\text{X}}}}$) [17–20]. The generic structure of the Eq. (1) that describes the process does not include any strain specific information and there are no any initial conditions assumed for the values of both $\alpha$ and $\beta$.

Simutis and Lübbert (2006) improved a hybrid model estimator [21]. The main improvement of a dynamical mathematical model was a modification of mass balance equation to the new one, which was based on the oxygen uptake rate OUR, the carbon dioxide rate CPR and the base consumption rate BCR [22]. In order to further improve hybrid model’s capacity, Kalman filter (EKF) was introduced to biomass estimations [23]. The new improved hybrid model produced better results and accuracy, but general drawbacks remained, estimator’s complexity, a lot of data required for artificial neural network training and biomass estimation offline with a large execution duration [22–24]. In 2010, Simutis and Lübbert improved biomass estimator with cumulative variables that made model more conventional. The estimator procedure was transformed to a simpler system.

**Fig. 1:**
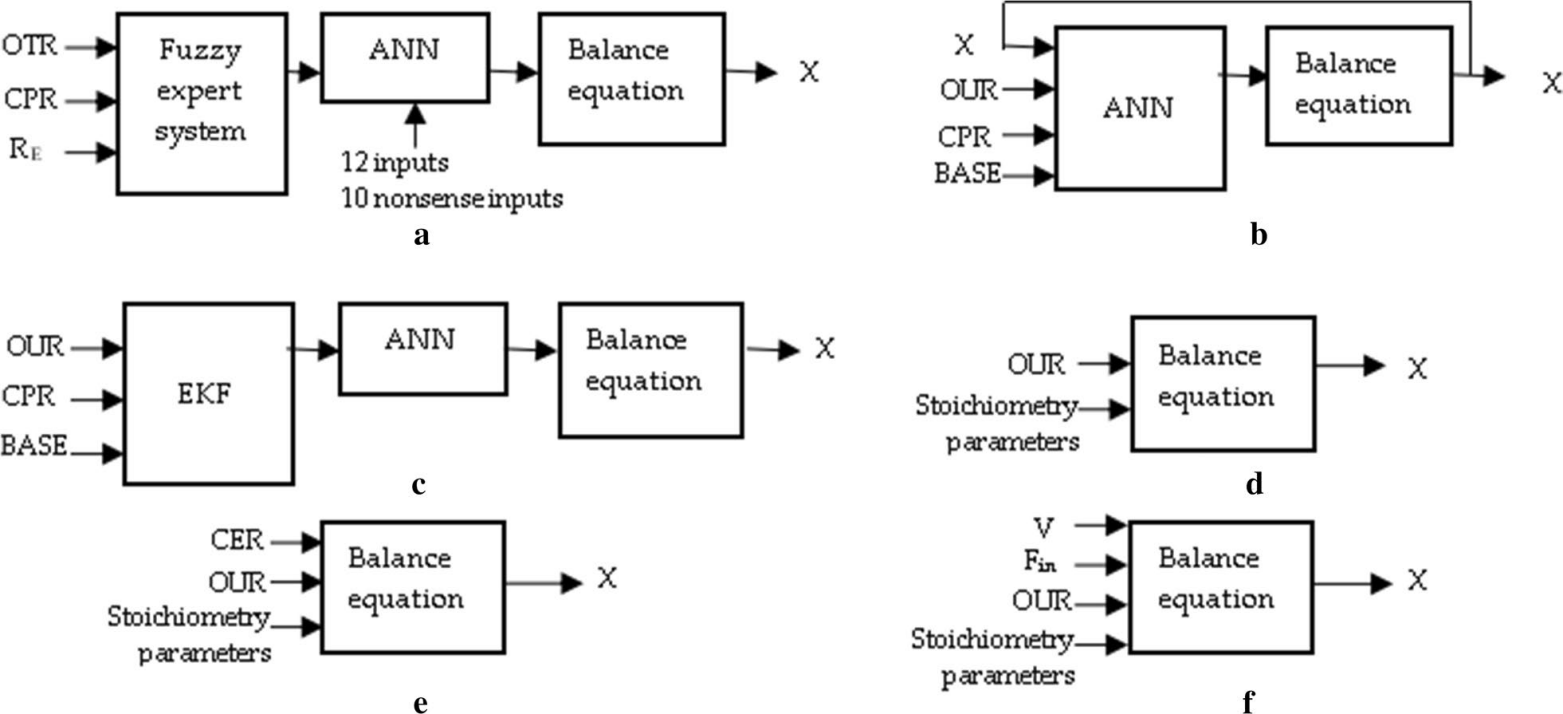
Comparison of biomass estimators: **a** Lübbert [21], **b** Achle [25], **c** Simutis [22], **d** biomass estimation of this text, **e** Davis [26], **f** Barrigon [27]

When comparing stoichiometry biomass estimators’ mathematical models to the hybrid model estimator approaches, the latter contains more main state variables: biomass (*X*), oxygen uptake rate (OUR), specific biomass growth rate (µ), broth weight (w), carbon dioxide production rate (CPR), base consumption and other model coefficients. Additionally, additional equations and a fuzzy expert system are required. The latter gives an input to the combination of a dynamical mathematical model (DMM) represented by a set of nonlinear ordinary differential equations with an artificial neural network (ANN) [24]. The main advantage of the stoichiometry biomass estimator, compared to hybrid model, is its simplicity and accuracy. As hybrid model consists of several modeling systems, a common problem of estimation arrives from artificial neural network (ANN) training [21, 23, 24]. Meanwhile, stoichiometry biomass estimator was based only on OUR and stoichiometric parameters *α* and *β*, which both were kept static for a particular cell strain. This led to ability to calculate biomass online [14, 22–24, 28]. A general comparison of different biomass estimators is presented in Fig. 1. This work’s biomass estimation approach is depicted by Fig. 1d. The estimation methods, which are based on gas consumption stoichiometry, are shown in Fig. 1e, f. The main differences consist of the approach picked, its complexity and the number of input signals and prerequisite parameters or initial conditions required. The main purpose of this paper is to show that biomass estimation can be treated from the fundamental point of view based on the stoichiometry Eq. (1). The idea comes from entropic and Bayesian inference approaches involving integral optimizations [29, 30]. The focus lays on the implementation, which can be not only used in scientific R&D laboratories, but also on the industrial plants level.

**Fig. 2:**
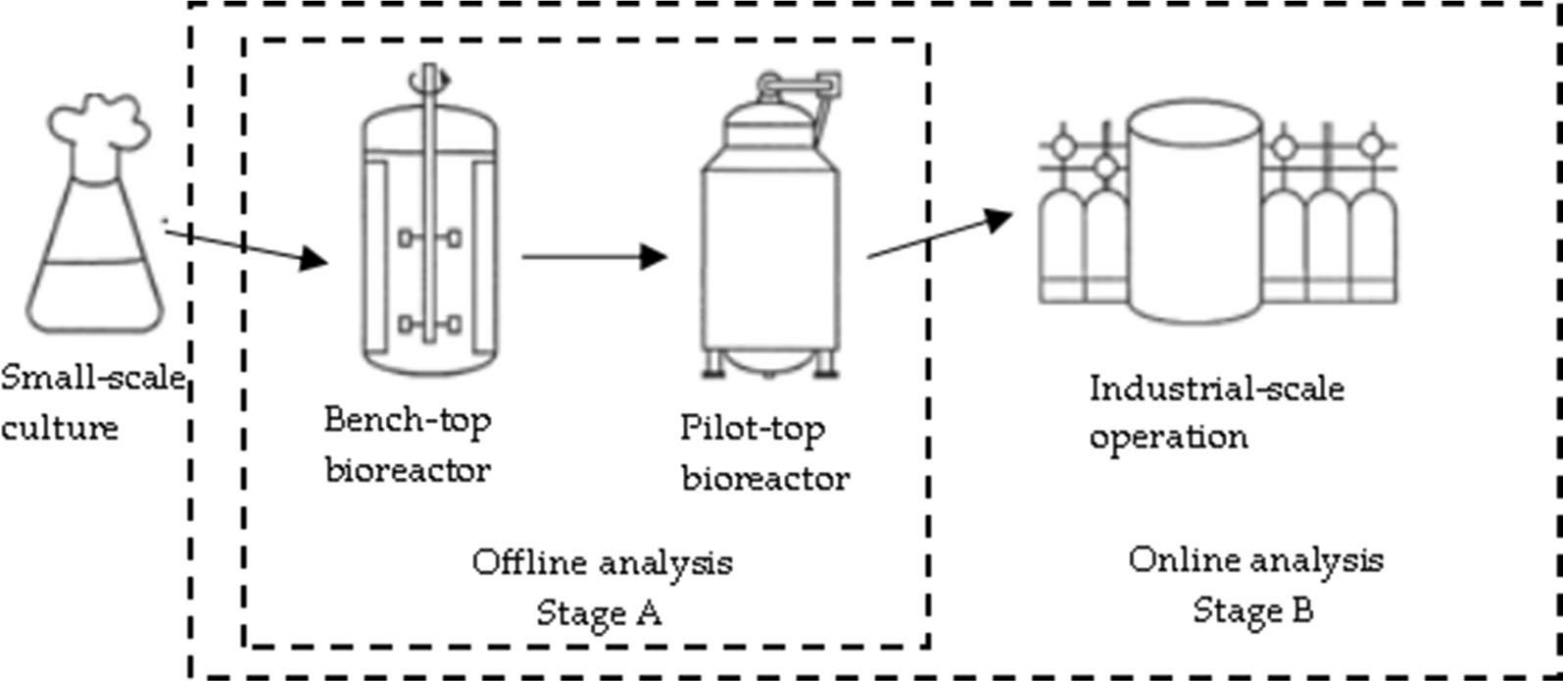
Bioprocess technology development workflow

**Fig. 3:**
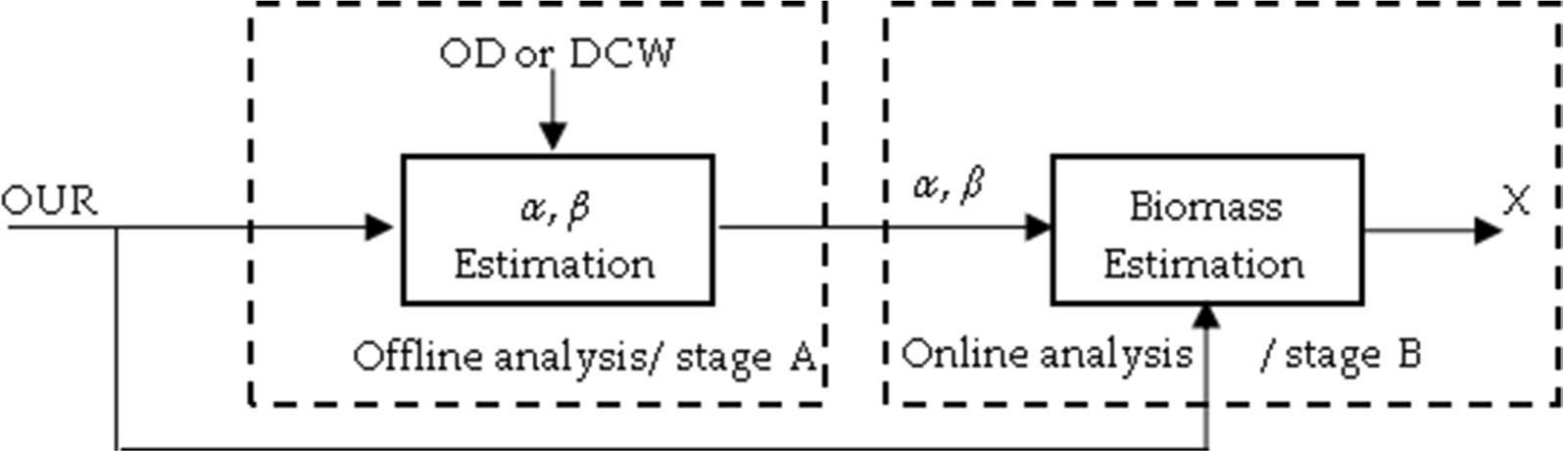
Biomass estimator’s structure scheme of different estimation stages

This paper presents a generic biomass estimation routine that is suitable for determination of biomass state in high diversity of bioreactors (Fig. 2) with potentially wide variety of industrial microorganisms. Prior to biomass determination, it is necessary to identify cell strain’s stoichiometry parameters *α* and *β*, which both describe oxygen consumption by a microbial culture. This is accomplished by offline analysis Fig. 3 (stage A).

Afterwards, industrial scale cultivation processes reuse information about strain information for corresponding biomass concentration estimation in online analysis (stage B), as shown in Fig. 3. In order to achieve better accuracy at strain stoichiometry analysis during upstream development, it is recommended to identify *α* and *β* parameters at the laboratory scale bioreactors, Fig. 3 (stage A). This way, strain stoichiometry analysis, based on “ground-truth” of stage A, is economically beneficial, and data from cultivation process consists of less disturbances in more flexible control environment.

## General mathematical model of stoichiometric parameters estimation

During the cultivation process, the real-time data collected from the devices has interference and disturbances, which may cause distortion of parameters and estimated values [14]. Simutis and Lübbert [4] stated “the reason for cumulating the original signals is to improve the signal-to-noise ratio (SNR) and thus increasing the information content about the process. Additionally, as the biomass and its metabolic products are accumulated during the cultivation, these masses are better correlated with the cumulative signals of OUR and CPR”. The main method of the current text is also based on the integral approach, which can be considered as a filter eliminating noise [22]. Hence, the Luedeking–Piret model Eq. (1) outcomes are being protected from disturbances by integrating it:

2 $$\mathop \int \nolimits_{{t_{0} }}^{t} OUR\left( {t^{*} } \right) {\text{d}}t^{*} = \alpha \cdot \mathop \int \nolimits_{{t_{0} }}^{t} X'\left( {t^{*} } \right){\text{d}}t^{*} + \beta \cdot \mathop \int \nolimits_{{t_{0} }}^{t} X\left( {t^{*} } \right) {\text{d}}t^{*} .$$

According to data from bioprocesses and previous experience, the stoichiometric parameter *β* is assumedly not a process constant. During the cultivation, parameter *β*—oxygen maintenance coefficient for biomass, increases due to biomass concentration growth. The phenomenon of increasing value of parameter *β* can be explained by the fact that the consumption of oxygen for biomass maintenance also includes the generation of the product and other factors. Such situation occurs at the end of the exponential phase of a microbial cultivation (for recombinant protein synthesis) when the induction (e.g., with isopropyl-d-1-thiogalactopyranoside/IPTG) is performed and the synthesis of the product increases noticeably. As a result, oxygen consumption for biomass maintenance also increases [31, 32]. The parameter *β* consists of two additive terms

3 $$\beta = \frac{1}{{Y_{XO} }} + \frac{1}{{Y_{PO} }};$$

where $Y_{XO}$ is oxygen consumption for cells breathing and $Y_{PO}$ is oxygen consumption for product formation. Consequently, biomass has linear/polynomial relationship to parameter β which is directly dependent on biomass concentration.

The observational data used for proposed biomass estimation was obtained from the processes that involve recombinant protein expression. As it can be seen from the Eq. (3), the parameter $\beta$ accounts for both, biomass and product, yields. This parameter may exhibit different behavior depending on the process phase and the strain/product involved. However, comprehensive comparison of various strains with respect to the impact, that particular product has on the biomass estimator performance, or to explore the effect on metabolic noise debugging in strain engineering, goes beyond the scope of this study.

To remove the assumption that the stoichiometric parameter β is a function of a biomass, this parameter is expressed as a function of time in the mathematical model. Hence, Eq. (3) is rewritten to linear regression of time:

4 $$\beta = k_{1} *t + k_{2} ;$$

where $k_{1}$ and $k_{2}$ are linearly dependent mathematical coefficients. When bioprocess is at lag phase or early phase of exponential growth (when biomass concentration is relatively low), the *β* parameter is extremely small and negligible. Only after induction or specific value of biomass concentration, oxygen consumption for maintenance becomes appreciable. Hence, during a time prior to fact when the Eq. (4) comes into effect, the parameter $\beta$ should be set to zero in the estimation procedure. At that moment the biomass concentration reaches a value from which the consumption of oxygen for biomass maintenance becomes significant:

5 $$0 = k_{1} *t_{i} + k_{2} \mathop{\longrightarrow}\limits^{yields}$$

6 $$k_{2} = - k_{1} * {\text{t}}_{\text{i}} .$$

Then parameter $\beta$ becomes

7 $$\beta = k_{1} *t - k_{1} *t_{i} ;$$

where $t_{i}$ is the duration from cultivation process start to the time when amount of biomass reaches value resulting in appreciable oxygen maintenance, or when induction is performed and product formation noticeably increases, or when stoichiometry parameter *β* is no longer zero [9, 31, 32]. In order to have full mathematical model formula, main balance Eq. (2) has parameter *β* replaced in the linear regression Eq. (7):

8 $$\mathop \int \nolimits_{{t_{0} }}^{t} OUR\left( {t^{*} } \right) {\text{d}}t^{*} = \alpha \cdot \mathop \int \nolimits_{{t_{0} }}^{t} X'\left( {t^{*} } \right){\text{d}}t^{*} + \mathop \int \nolimits_{{t_{0} }}^{t} k_{1} \cdot \left( {t^{*} - t_{i} } \right) \cdot X\left( {t^{*} } \right) {\text{d}}t^{*} .$$

### Offline analysis of stoichiometry parameters (stage A)

Prior to the estimation of the biomass, specific cell strain’s stoichiometric parameters must be identified during offline analysis. There are few compulsory inputs to approach this task.

- Model fitting procedure requires offline observations: dry cell weight (DCW) or optical density OD value (in o.u.) multiplied by a coefficient of biomass concentration (approximately 0.4 g/l/o.u.) [33];
- Process duration time since cells’ inoculation to bioreactor, in hours;
- Oxygen uptake rate (OUR) data since the inoculation;

For model fitting a chosen mathematical expression is equated to gray box model since the collected experimental data is combined with fundamental knowledge about bioprocess [34]. Considering that the bioprocess consists of two main parts, prior to induction and after it, the parameters fitting procedure is based on two independent gray box models. The first one covers the first two cultivation process phases: the lag and exponential. During these phases the amount of biomass is low and materials, resources concentrate to biomass growth [35]. Hence, oxygen requirement for biomass maintenance is minimum and stoichiometric parameter *β* is negligible:

9 $$\mathop \int \nolimits_{{t_{0} }}^{{t_{i} }} OUR\left( {t^{*} } \right) {\text{d}}t^{*} = \alpha \cdot \mathop \int \nolimits_{{t_{0} }}^{{t_{i} }} X'\left( {t^{*} } \right){\text{d}}t^{*} ,$$

In the Eq. (9) the variable $t_{i}$ is the time of the induction or the time when biomass reaches a quantity where oxygen usage for maintenance is appreciable. The second cultivation stage represents the biomass growth deceleration and increasing product formation. In this cultivation phase, additional term comes into effect, oxygen consumption for maintenance and product formation, known as stoichiometric parameter *β*. To properly describe second gray box model, the induction time or time when biomass concentration reaches specific amount must be identified. Throughout this period the maintenance term becomes significant and can’t be negligible. After applying maintenance parameter to a model, the second gray box model’s expression is generalized to

10 $$\mathop \int \nolimits_{{t_{0} }}^{t} OUR\left( {t^{*} } \right) {\text{d}}t^{*} = \alpha \cdot \mathop \int \nolimits_{{t_{0} }}^{t} X'\left( {t^{*} } \right){\text{d}}t^{*} + \mathop \int \nolimits_{{t_{0} }}^{t} k_{1} \cdot \left( {t^{*} - t_{i} } \right) \cdot X\left( {t^{*} } \right) {\text{d}}t^{*} .$$

In summary, the Eqs. (10) and (11) both yield the conditional definition of cumulative oxygen uptake rate function:

11 $$\left\{ {\begin{array}{*{20}l} {cOUR\left( {t \le t_{i} } \right) \equiv \mathop \int \nolimits_{{t_{0} }}^{t} OUR\left( {t^{*} } \right) {\text{d}}t^{*} = \alpha \cdot \left( {X\left( t \right) - X_{0} } \right); \quad t \le t_{i} ;} \\ {cOUR\left( {t > t_{i} } \right) \equiv \mathop \int \nolimits_{{t_{0} }}^{t} OUR\left( {t^{*} } \right) {\text{d}}t^{*} \approx \alpha \cdot \left( {X\left( t \right) - X_{0} } \right) + \mathop \sum \nolimits_{l = i}^{m} k_{1} \cdot \left( {t_{l} - t_{i} } \right) \cdot X\left( {t_{l} } \right) \cdot \Delta t_{l,l - 1} ; \quad t > t_{i} .} \\ \end{array} } \right.$$

In Eq. (11) the last sum of products is the expression of left Riemann sum [36], i.e. $\mathop \smallint \limits_{{t_{0} }}^{t} k_{1} \cdot \left( {t^{*} - t_{i} } \right) \cdot X\left( {t^{*} } \right) {\text{d}}t^{*} \approx \mathop \sum \nolimits_{l = i}^{m} k_{1} \cdot \left( {t_{l} - t_{i} } \right) \cdot X\left( {t_{l} } \right) \cdot \Delta t_{l,l - 1}$, when time’s *t* sample is indexed by *m*. Discrete DCW values define variable $X_{l} \equiv X\left( {t_{l} } \right), {\text{where}}\; l \in \left[ {1,n_{m} } \right]$, $n_{m}$ is the total number (e.g. hourly) of offline sampling intervals with index *m* and $X_{0} \equiv X\left( {t_{0} } \right)$ is an initial biomass concentration after inoculation into bioreactor.

#### Procedure for offline analysis of stoichiometry parameters

The prediction value of the cumulative OUR model [37] for Eq. (11) is

12 $$cOUR_{m} \equiv \left\{ {\begin{array}{*{20}l} {cOUR\left( {t_{m} \le t_{i} } \right) = \alpha \cdot \left( {X_{m} - X_{0} } \right);} \\ {cOUR\left( {t_{m} > t_{i} } \right) = \alpha \cdot \left( {X_{m} - X_{0} } \right) + \mathop \sum \nolimits_{l = i}^{m} k_{1} \cdot \left( {t_{l} - t_{i} } \right) \cdot X_{l} \cdot \Delta t_{l,l - 1} ;} \\ \end{array} } \right.$$

Then the posterior distribution for *m*-th offline sample is

13 $$P_{posterior} \left( {cOUR_{m} } \right)\sim N\left( {cOUR_{m} ,\sigma_{cOUR}^{2} } \right),$$

where every sampled prediction *m* has constant variance $\sigma_{cOUR}^{2}$.

Prior distribution also has the form of Gaussian distribution [38]

14 $$P_{likelihood} \left( {cOUR_{m} } \right)\sim N\left( {cOUR_{m}^{*} ,\sigma_{cOUR,m}^{2} } \right),$$

where $cOUR_{m}^{*}$ is the *m*-th observation value of the cumulative OUR and its unique variance is $\sigma_{cOUR,m}^{2}$.

In previous work [37] the uncertainty of prior distribution was assumed to be equal to the square of observed value, i.e. $\sigma_{cOUR,m}^{2}$ was assumed to be proportional to $cOUR_{m}^{*2}$. However, this assumption is not quite rational from practical considerations based on this work experience when deriving a generic estimator for both *E. coli* and yeast cultures. It appears that the assumption of $\sigma_{cOUR,m}^{2} \sim cOUR_{m}^{*2}$ is just a special case, which has even more general form. Interestingly this form matches the form of Monod formulation [39] applied to uncertainty, i.e.

15 $$\sigma_{cOUR,m}^{2} = \sigma_{max}^{2} \frac{{X_{m}^{2} }}{{K_{{X^{2} }} + X_{m}^{2} }},$$

where scenario with $K_{{X^{2} }} = 0$ resembles least squares approach, i.e. all samples’ relative weights become equal, and $K_{{X^{2} }} \to \infty$ means that $\sigma_{cOUR,m}^{2} \sim cOUR_{m}^{*2}$ as in previous work [37]. In other words, empirical coefficient $K_{{X^{2} }}$ is a “weight” coefficient between the two additive terms of optimization criterion. The first term is the least squares criterion and the other is “squared MAPE” criterion as in [37]. Another note about Monod Eqs. (15) and (12) is that the relationship of $\sigma_{cOUR,m}^{2} \sim \sigma_{X,m}^{2}$ is valid, i.e. the uncertainty of cumulative OUR is proportional to the uncertainty of biomass variable.

To rationally prepare Eq. (15) for simplified numeric operations avoiding infinities when estimating values, an intrinsic variable $K_{exp}$ expression replaces $K_{{X^{2} }} \to \frac{{1 - K_{exp} }}{{K_{exp} }}$ and transforms Eq. (15) to

16 $$\sigma_{cOUR,m}^{2} = \sigma_{max}^{2} \frac{{X_{m}^{2} }}{{\frac{{1 - K_{exp} }}{{K_{exp} }} + X_{m}^{2} }}\mathop{\longrightarrow}\limits^{yields}\sigma_{max}^{2} \frac{{X_{m}^{2} \cdot K_{exp} }}{{1 - K_{exp} + X_{m}^{2} \cdot K_{exp} }},$$

The fact, that $\sigma_{max}^{2}$ and $K_{exp}$ both are positive scalar values and do not depend on the index *m* of a sampling interval, allows to simplify Eq. (16) to

17 $$\sigma_{cOUR,m}^{2} \sim \frac{{X_{m}^{2} }}{{1 - K_{exp} + X_{m}^{2} \cdot K_{exp} }}.$$

Equation (17) exposes the physical meaning of $K_{exp}$. The scenario with $K_{exp} = 0$ recovers $\sigma_{cOUR,m}^{2} \sim X_{m}^{2}$ as in [37]. The scenario with $K_{exp} = 1$ recreates the least squares method as in [38, 40]. Both scenarios show that $K_{exp}$ is an exponential weight, which constructs a hybrid criterion for both least squares and the MAPE squared. Later in the text, the experimental validation will show that there exists a rational empirical value of $K_{exp}$, which enables estimation of the biomass concentration, with an acceptable precision, for both yeast and *E. coli* cultures since the beginning of the cultivation right after the culture was inoculated to a bioreactor.

After gray box model is identified and hybrid criterion derived, the next step is to use optimization approach to find the stoichiometry parameters. The main equation solving for unknown parameters comes from the maximization of entropy [37, 39] based on Eqs. (13), (14) and (17)

18 $$\begin{aligned} S & = - \mathop \sum \limits_{m = 1}^{m \le i} \frac{{\left( {cOUR_{m} - \alpha \cdot \left( {X_{m} - X_{0} } \right)} \right)}}{{\frac{{X_{m}^{2} }}{{K_{exp} \cdot X_{m}^{2} + \left( {1 - K_{exp} } \right)}}}}^{2} \\ & \quad - \mathop \sum \limits_{m = i + 1}^{{n_{m} }} \frac{{\left( {cOUR_{m} - \alpha \cdot \left( {X_{m} - X_{0} } \right) - \mathop \sum \nolimits_{l = 1}^{m} k_{1} \cdot \left( {t_{l} - t_{i} } \right) \cdot X\left( {t_{l} } \right) \cdot \Delta t_{l,l - 1} } \right)^{2} }}{{\frac{{X_{m}^{2} }}{{K_{exp} \cdot X_{m}^{2} + \left( {1 - K_{exp} } \right)}}}}. \\ \end{aligned}$$

Hence, at the optimization method, which is shown at the Eq. (18), the whole *S* expression is maximized, and unknown stoichiometry parameters are found by solving partial derivative of Eq. (18) with respect to α and *k*_*1*_

19 $$\left\{ {\begin{array}{*{20}c} {\frac{\partial S}{\partial \alpha } = 0;} \\ {\frac{\partial S}{{\partial k_{1} }} = 0.} \\ \end{array} } \right.$$

Equation (19) yields the linear system of two equations

20 $$\left\{ {\begin{array}{*{20}c} {\alpha \cdot B + k_{1} \cdot C = A;} \\ {\alpha \cdot E + k_{1} \cdot F = D;} \\ \end{array} } \right..$$

where Eq. (20) parameters are:

21 $$A = \mathop \sum \limits_{m = 1}^{{n_{m} }} \frac{{\left( {X_{m} - {\text{X}}_{0} } \right) \cdot cOUR_{m} }}{{\frac{{X_{m}^{2} }}{{K_{exp} \cdot X_{m}^{2} + \left( {1 - K_{exp} } \right)}}}};$$

22 $$B = \mathop \sum \limits_{m = 1}^{{n_{m} }} \frac{{\left( {X_{m} - X_{0} } \right)^{2} }}{{\frac{{X_{m}^{2} }}{{K_{exp} \cdot X_{m}^{2} + \left( {1 - K_{exp} } \right)}}}};$$

23 $$C = \mathop \sum \limits_{m = i + 1}^{{n_{m} }} \frac{{\left( {X_{m} - X_{0} } \right) \cdot \mathop \sum \nolimits_{l = 1}^{m} \left( {t_{l} - t_{i} } \right) \cdot X\left( {t_{l} } \right) \cdot \Delta t_{l,l - 1} }}{{\frac{{X_{m}^{2} }}{{K_{exp} \cdot X_{m}^{2} + \left( {1 - K_{exp} } \right)}}}};$$

24 $$D = \mathop \sum \limits_{m = i + 1}^{{n_{m} }} \frac{{cOUR_{m} \cdot \mathop \sum \nolimits_{l = 1}^{m} \left( {t_{l} - t_{i} } \right) \cdot X_{l} \cdot \Delta t_{l,l - 1} }}{{\frac{{X_{m}^{2} }}{{K_{exp} \cdot X_{m}^{2} + \left( {1 - K_{exp} } \right)}}}};$$

25 $$E = \mathop \sum \limits_{m = i + 1}^{{n_{m} }} \frac{{\left( {X_{m} - X_{0} } \right) \cdot \mathop \sum \nolimits_{l = 1}^{m} \left( {t_{l} - t_{i} } \right) \cdot X_{l} \cdot \Delta t_{l,l - 1} }}{{\frac{{X_{m}^{2} }}{{K_{exp} \cdot X_{m}^{2} + \left( {1 - K_{exp} } \right)}}}};$$

26 $$F = \mathop \sum \limits_{m = i + 1}^{{n_{m} }} \frac{{\left( {\mathop \sum \nolimits_{l = 1}^{m} \left( {t_{l} - t_{i} } \right) \cdot X_{l} \cdot \Delta t_{l,l - 1} } \right)^{2} }}{{\frac{{X_{m}^{2} }}{{K_{exp} \cdot X_{m}^{2} + \left( {1 - K_{exp} } \right)}}}}.$$

Equations (20)–(26) finalizes the offline estimation of stoichiometry parameters, which are then later used for online estimation of biomass concentration. However, the variable $t_{i}$ has no direct meaning with yeast cultures, so it must be dealt with separately. First, the specific time when the maintenance coefficient becomes appreciable is analyzed in the next subsection.

#### Identification of yeasts’ specific time for maintenance

**Fig. 4:**
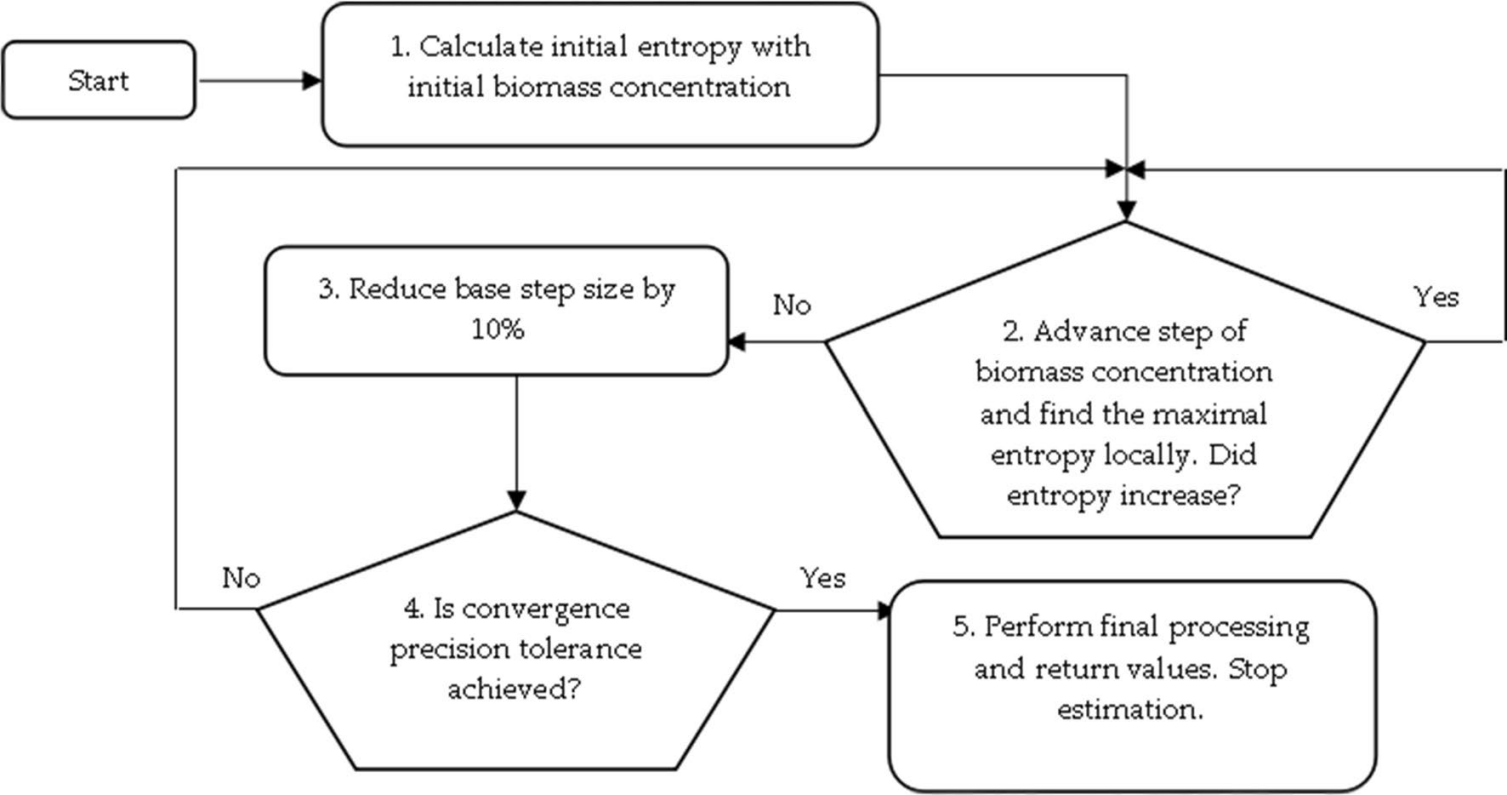
The workflow of structural scheme for parameter identification

Variable $t_{i}$ at Eq. (12) is the time of induction or the time when biomass concentration reaches a specific amount when oxygen maintenance for cells becomes non negligible. In the case of cultivation processes of *E. coli*, the induction time is known, i.e. it can be defined by the time moment when IPTG solution is injected into bioreactor. In the cultivation process of *S. cerevisiae* yeasts the IPTG solution was not used. Hence, the variable $t_{i}$ defines the time when biomass concentration reaches a specific value when maintenance coefficient becomes noticeable. The search for $t_{i}$ utilizes the convex optimization method and maximization of entropy [37, 41]. The optimization procedure is depicted in Fig. 4.

The knowledge of the specific time $t_{i}$ enables the biomass concentration estimation. However, the specific time $t_{i}$ is not known in advance prior to online experiment with yeast cells, because it has just a theoretical meaning in this case. Therefore, a generic relationship between the maintenance coefficient value and the biomass concentration will be inferred in the next subsection. Such a generic form of maintenance coefficient will enforce online estimation without dependence on the type of the microbial culture. Moreover, the value of the specific time $t_{i}$ becomes irrelevant for the online estimation procedure.

#### Identification of maintenance coefficient parts

After optimization of stoichiometry parameters, which had determined unknown parameters of the mathematical method, the next step is to validate those identified parameters with experimental data. Prior to comparison of theoretical and experimental data, the mathematical model, as in Eq. (7), must be reconstructed so that $\beta$ is no longer a function of time and still satisfies the actual behavior of biotechnological process. The stoichiometric parameter *β* directly depends on biomass concentration

27 $$\beta \left( X \right) \equiv \beta \left( {X\left( t \right)} \right) = k_{\beta 2} \cdot X^{2} \left( t \right) + k_{\beta 1} \cdot X\left( t \right) + k_{\beta 0} ;$$

**Fig. 5:**
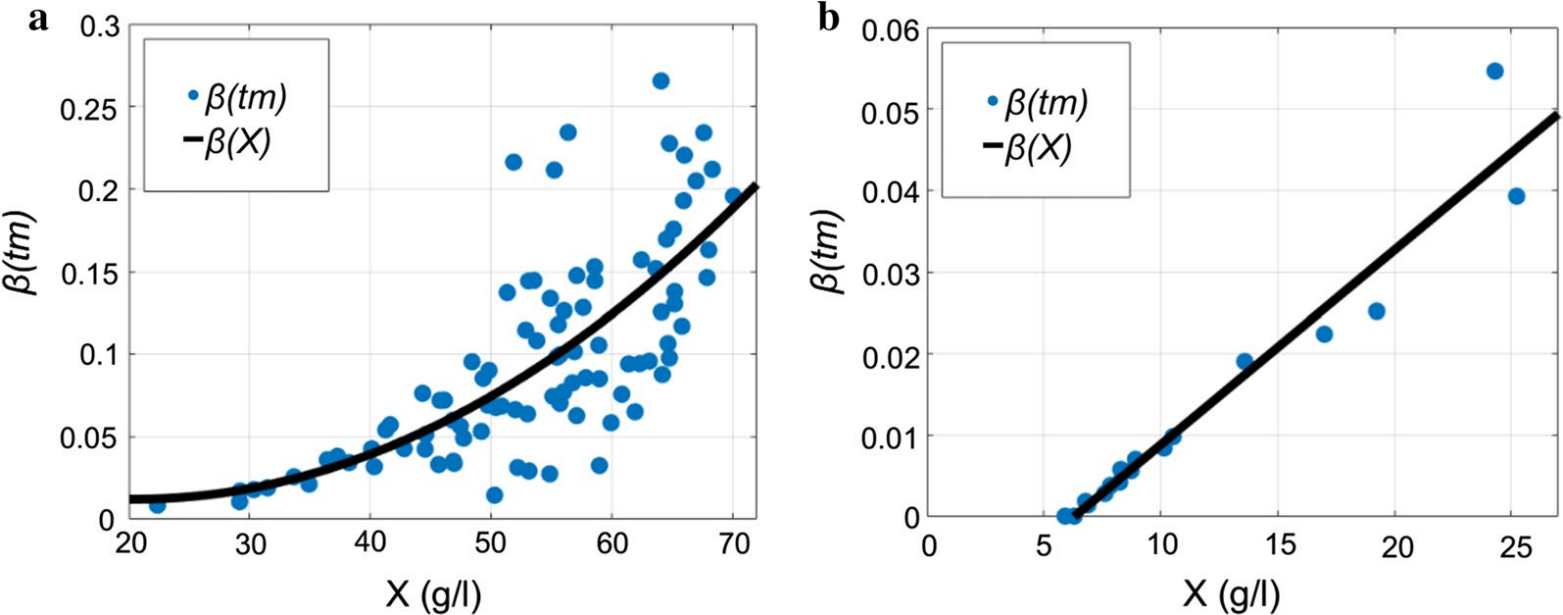
Dependence of oxygen consumption for maintenance on biomass concentration, **a** *E. coli*, **b** *Saccharomyces cerevisiae*

The expression of parameter $\beta \left( X \right)$ represents a parabola regression of biomass in the case of the *E. coli* strain Fig. 5a. Meanwhile, *S. cerevisiae* oxygen consumption for maintenance is dependent linearly on biomass concentration, thus $k_{\beta s2} = 0$,

28 $$\beta_{Saccharomyces} \left( X \right) \equiv \beta_{Saccharomyces} \left( {X\left( t \right)} \right) = k_{\beta s1} \cdot X\left( t \right) + k_{\beta s0} ;$$

In Eqs. (27) and (28) regression coefficients connect maintenance coefficient $\beta$ to biomass variable. In both culture cases, stage A helps to obtain *β* values from linear regression based on Eq. (7) output

29 $$\beta \left( {X_{m} } \right) \cong \beta \left( {t_{m} } \right) = k_{1} *\left( {t_{m} - t_{i} } \right).$$

The assumed relationship of $\beta \left( X \right)$ considering biomass concentration is presented in Fig. 5.

According to data from cultivation processes of *E. coli* in Fig. 5, the stoichiometric parameter of cell maintenance can be assumed as directly dependent on biomass in parabolic manner. At the cultivation processes of *E. coli*, the induction of IPTG, which initiates product synthesis, may cause nonlinear dependence of oxygen consumption on biomass maintenance. Based on Eqs. (27) and (28), it is possible to calculate strain’s specific biomass concentration ($X_{specific}$) when oxygen consumption for maintenance is no longer negligible. This is done by setting Eqs. (27) and (28) to zero and solving them for the specific biomass concentration $X_{specific}$

30 $$\beta \left( {X_{specific} } \right) \equiv \beta \left( {X\left( t \right)} \right) = 0;$$

**Fig. 6:**
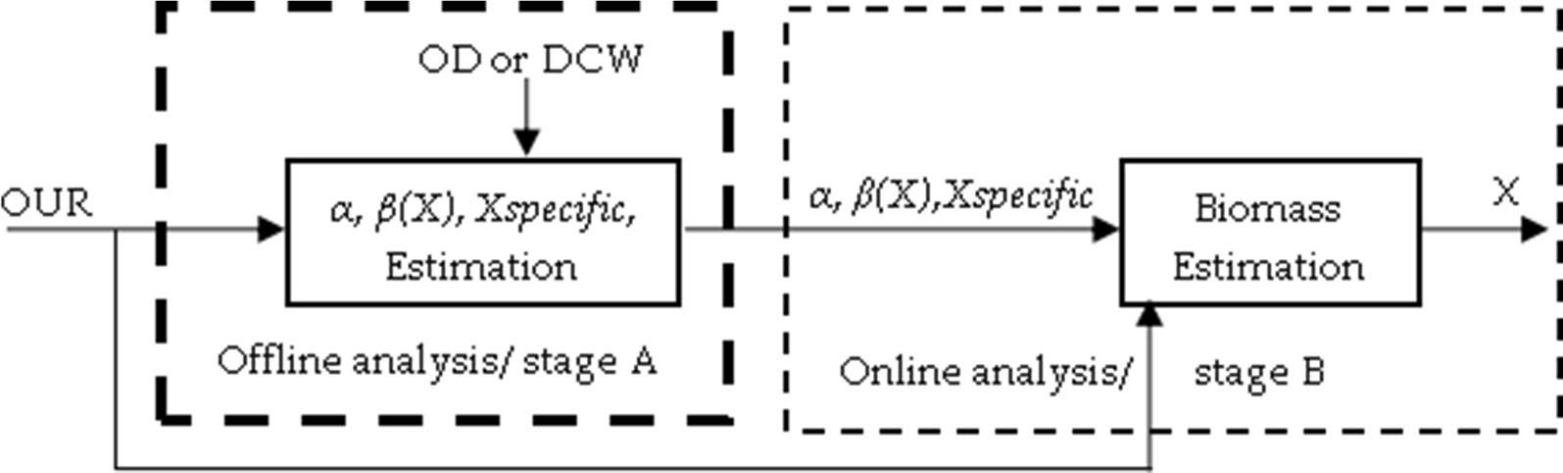
Biomass estimation workflow

The workflow of both stoichiometry and biomass estimations improves structure, as in Fig. 3, to the shape of the one in Fig. 6.

The solution of Eq. (30) identifies the specific biomass concentration $X_{specific}$ and finalizes the offline estimation of stoichiometry coefficients for a strain. After the stoichiometry coefficients are found in stage A, a generic procedure for online biomass estimation can be performed independently on the knowledge of bioreactor parameters. In conclusion, $\beta$, as in Eq. (27), transforms Eq. (1) into

31 $$\left\{ {\begin{array}{*{20}l} {OUR\left( t \right) = \alpha \cdot X^{\prime}\left( t \right) + k_{\beta 2} \cdot X^{3} \left( t \right) + k_{\beta 1} \cdot X^{2} \left( t \right) + k_{\beta 0} \cdot X\left( t \right),X\left( t \right) > X_{specific} ;} \\ {OUR\left( t \right) = \alpha \cdot X^{\prime}\left( t \right),X\left( t \right) \le X_{specific} .} \\ \end{array} } \right.$$

In spite of the fact that Eq. (31) form is the third order function, it is still the same equation as Eq. (1). However, it was inferred by the estimation procedure and the observation data in Fig. 5. Variable $\beta$ manipulation compensates the effect of biomass concentration *X* on $\beta$ and makes all Eq. (31) coefficients linearly dependent and constant throughout the course of the experiment. Eventually, this serves as a prerequisite to the simplified generic procedure for estimation of biomass concentration, coming in the next subsection.

### Online estimation of biomass concentration (stage B)

In this paper, estimation of biomass concentration is based on stoichiometric parameters and cumulative oxygen uptake rate *cOUR*. When stoichiometric parameters are discovered in stoichiometry estimation, stage A, or it was given, only one input from bioreactor system, cumulative oxygen uptake rate, is necessary to estimate the biomass state. This procedure is depicted by stage B (online analysis) in Fig. 6. The block of “biomass estimation”, Fig. 6, consists of two main scenarios which both return biomass concentration at a time instance with index *m*. Prior to the specific biomass $X_{specific}$ level is reached, i.e. when oxygen consumption for maintenance is very low or negligible, biomass state estimator equation is

32 $$X_{m} = \frac{{cOUR_{m} }}{\alpha } + X_{0} .$$

After biomass concentration exceeds $X_{specific}$ during the second scenario, i.e. oxygen consumption becomes noticeable, the stoichiometric parameter *β* comes into effect as a function of biomass concentration. Equation (12) helps to derive the approximate estimator for biomass state, as follows

33 $$X_{m} \cong \frac{{cOUR_{m} - \mathop \sum \nolimits_{l = i}^{m - 1} \beta \left( {X_{l} } \right) \cdot X_{l} \cdot \Delta t_{l,l - 1} }}{\alpha } + X_{0} .$$

The variable $X_{0}$, as in Eqs. (32) and (33), is an initial biomass concentration at the time of inoculation into bioreactor. Its value can be either a dry biomass measurement value or optical density OD value (in o.u.) multiplied by a coefficient of biomass concentration (approximately 0.4 g/l/o.u.).

**Fig. 7:**
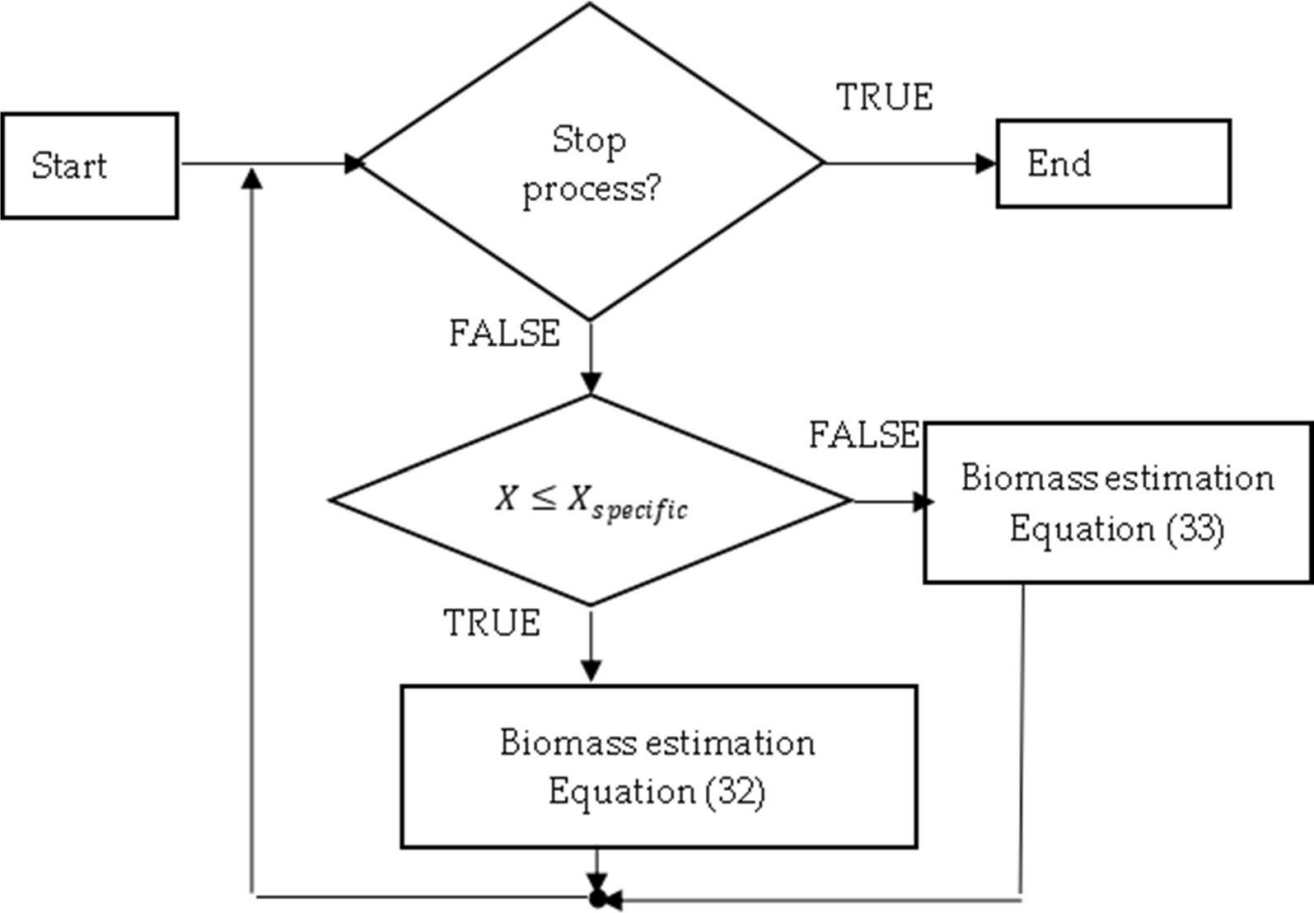
Workflow of online biomass estimation (stage B)

This subsection initializes the online biomass estimation procedure (Fig. 7), which can be used in biotechnological industrial practices. The suggested approach does not require the bioreactor-dependent parameters, it serves as a good candidate to be applied to more microbial strains and the experimental validation, in the coming section, will show that such an approach can be used for biomass estimation since the time moment of inoculation into bioreactor.

## Experimental validation

### Validation performance indicators

Both mean absolute error (MAE) and mean absolute percentage error (MAPE) were used as indicators to evaluate the estimation results. MAE and MAPE methods both evaluate the errors between estimated and observed biomass values of a cultivation process. MAE approach is defined as follows [42]:

34 $$MAE = \frac{{\mathop \sum \nolimits_{i = 1}^{n} \left| {\hat{y}_{i} - y_{i} } \right|}}{n} ,$$

where *n* is the number of data counts, $\hat{y}_{i}$ is estimation result, which is compared to $y_{i}$, the observed value from the cultivation process. Mean absolute error represents average vertical distance between both values. MAPE method can be expressed as follows [43]:

35 $$MAPE = \frac{100 \% }{n}\mathop \sum \limits_{i = 1}^{n} \left| {\frac{{\hat{y}_{i} - y_{i} }}{{y_{i} }}} \right|.$$

The mean absolute percentage error is a statistical measure representing the accuracy of a forecast system, in percentage. Root mean square error represents the square root of residuals of the differences between predicted values and observed values. RMSE method’s formula are as follows [42]:

36 $$RMSE = \sqrt {\frac{{\mathop \sum \nolimits_{i = 1}^{n} \left( {\hat{y}_{i} - y_{i} } \right)^{2} }}{n}} .$$

### Comparative analysis of experimental results

Experimental biomass measurements and data of cumulative oxygen uptake rate *cOUR* from fed-batch experiments of *E. coli* and *S. cerevisiae* were taken from [8], experiments led by authors of this text and industrial R&D laboratories. There were three cultivations of *E. coli* cells in 15 l bioreactor with limited substrate feed [8] and two R&D laboratory cultivations of *S. cerevisiae* yeasts in 5 l bioreactor with limited substrate feed. Additionally, there was one cultivation of *E. coli* in 12 l bioreactor with limited substrate feed and there were 15 cultivations in 5 l bioreactor, out of which 7 cultivations were with dosed substrate feeding. As the first step, all cultivation data was analyzed in the stoichiometric parameters’ estimation (stage A). The estimation procedure ignored both metabolism pathways, occurring during dosed substrate feed cultivations, and increasing product formation due to IPTG injections. The results of offline analysis of stoichiometric parameters are present in Table 1.

**Table 1:** Stoichiometric parameters of cell strains

*Escherichia coli*	*Saccharomyces cerevisiae*
*α* = 1.01	*α* = 1.35
Confidence Interval $\mp 0.0186$	Confidence Interval $\mp 0.149$
$k_{\beta e2} = 7.2 \cdot 10^{ - 5}$	$k_{\beta s2} = 0$
$k_{\beta e1} = - 2.9625 \cdot 10^{ - 3}$	$k_{\beta s1} = 2.3851 \cdot 10^{ - 3}$
$k_{\beta e0} = 4.27047{\text{d }} \cdot 10^{ - 2}$	$k_{\beta s0} = - 1.5014 \cdot 10^{ - 2}$
$X_{specific} = 20.6 {\text{g}}/{\text{l}}$	$X_{specific} = 6.29 {\text{g}}/{\text{l}}$
$$K_{exp} = 0.4$$	

The tuning coefficient $K_{exp}$ was identified empirically and its value of 0.4 showed acceptable outcome for the performed experiments. However, *S. cerevisiae* stoichiometric results come from just two cultivation experiments. Therefore, the results might still be improved when more experimental data becomes available in the future.

In industrial processes, strain’s stoichiometric parameters are given, unless they were estimated using offline analysis, stage A. Then biomass concentration is calculated iteratively using both Eqs. (32) and (33) from *cOUR* signal (online analysis, stage B). This work’s biomass estimation method used different cultivation experiments, with different cell strains, bioreactor volumes, type of substrates feeding solution, different IPTG induction time moment and their corresponding OD levels at IPTG injection, different substrate feeding limitations and different time of starting the substrate feed. Estimation results are shown in Table 2.

**Table 2:** Analysis of experiments for biomass estimation

No.	Strain	Bioreactor volume, l	MAE since inoculation g/l	MAE since feed start, g/l	MAPE since inoculation, %	MAPE since feed start, %
1	*Escherichia coli*	15	1.04	1.04	5.7	5.7
2			0.96	0.96	4.11	4.11
3			1.38	1.38	5.61	5.61
4		12	1.45	1.6	5.3	4.91
5		7	1.65	2.3	7.26	5.34
6			2.5	3.3	6.9	7
7			1.54	2.13	8.67	6.28
8			1.29	1.94	7.57	6.12
9			1.52	2.54	7.12	7.96
10			1.55	2.09	9.35	6
11			1.87	2.7	10.75	6.6
12			1.16	1.55	6.88	6.61
13			0.97	1.22	9.03	6.89
14			0.78	1.01	9.88	6.26
15			1	1.22	8.92	6.99
16			0.76	1	7.14	7.85
17			0.74	0.9	8.51	5.69
18			1.18	1.54	7.83	7.55
19			0.8	1.07	5.83	6.30
20	Yeasts	5	0.29	0.29	6.69	6.69
21			0.66	0.66	7.28	7.28

Seven experiments (#5–#11) were performed with dosed substrate feeding. Meanwhile the rest of experiments had limited feeding with various combinations of control strategies described in [37]: multiple different substrate limited feedings prior to induction and after it.

**Fig. 8:**
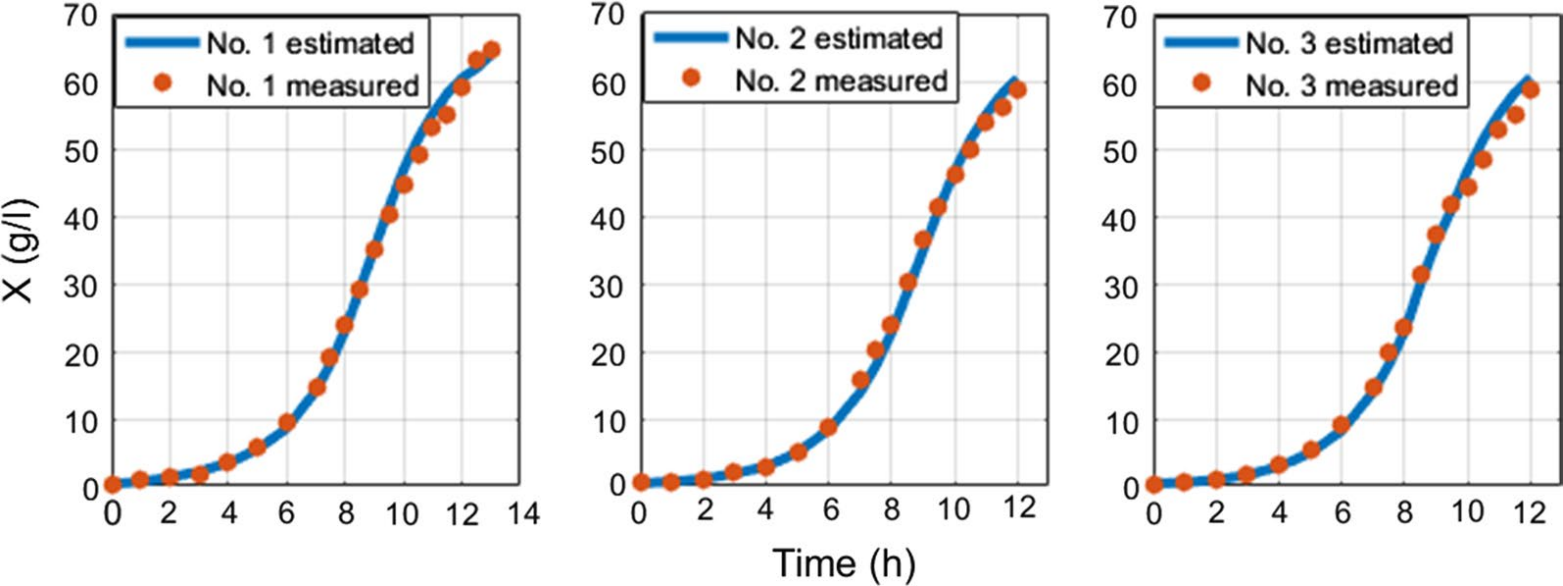
Biomass concentration estimation result with recombinant *E.coli* BL21(DE3) pLysS strain at 15 L bioreactor

**Fig. 9:**
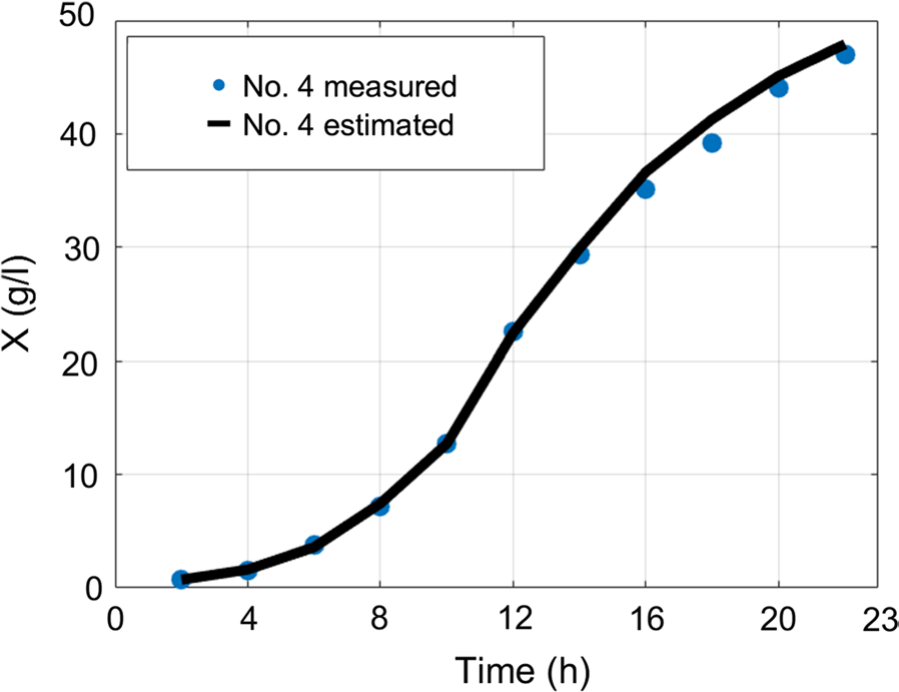
Biomass concentration estimation result with recombinant *E. coli* BL21(DE3) pET9a-IdeS strain at 12 l bioreactor

**Fig. 10:**
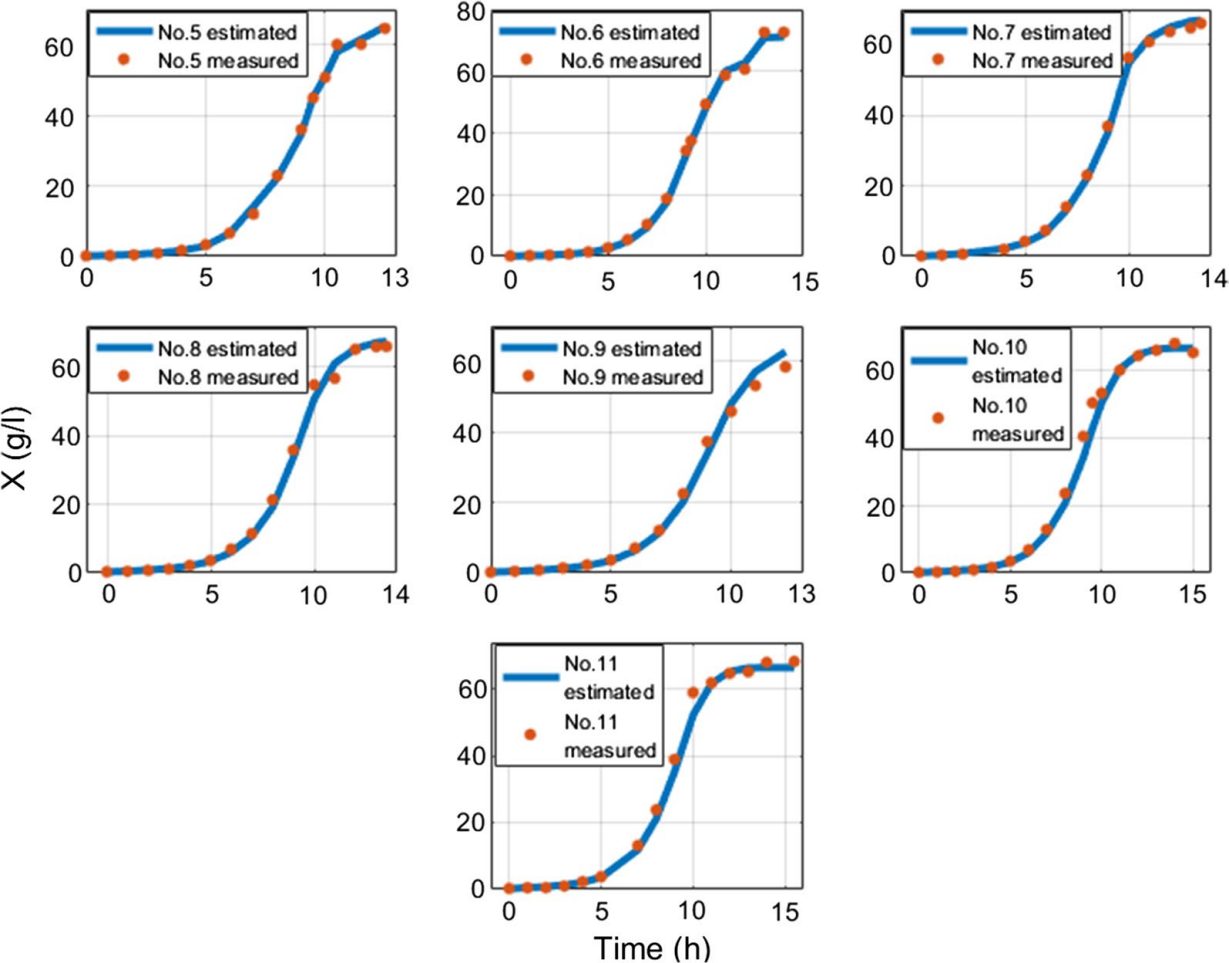
Biomass concentration estimation result with recombinant *E. coli* BL21 (DE3) pET21-IFN-alfa-5 strain at 7 L bioreactor with dosed substrate feeding

**Fig. 11:**
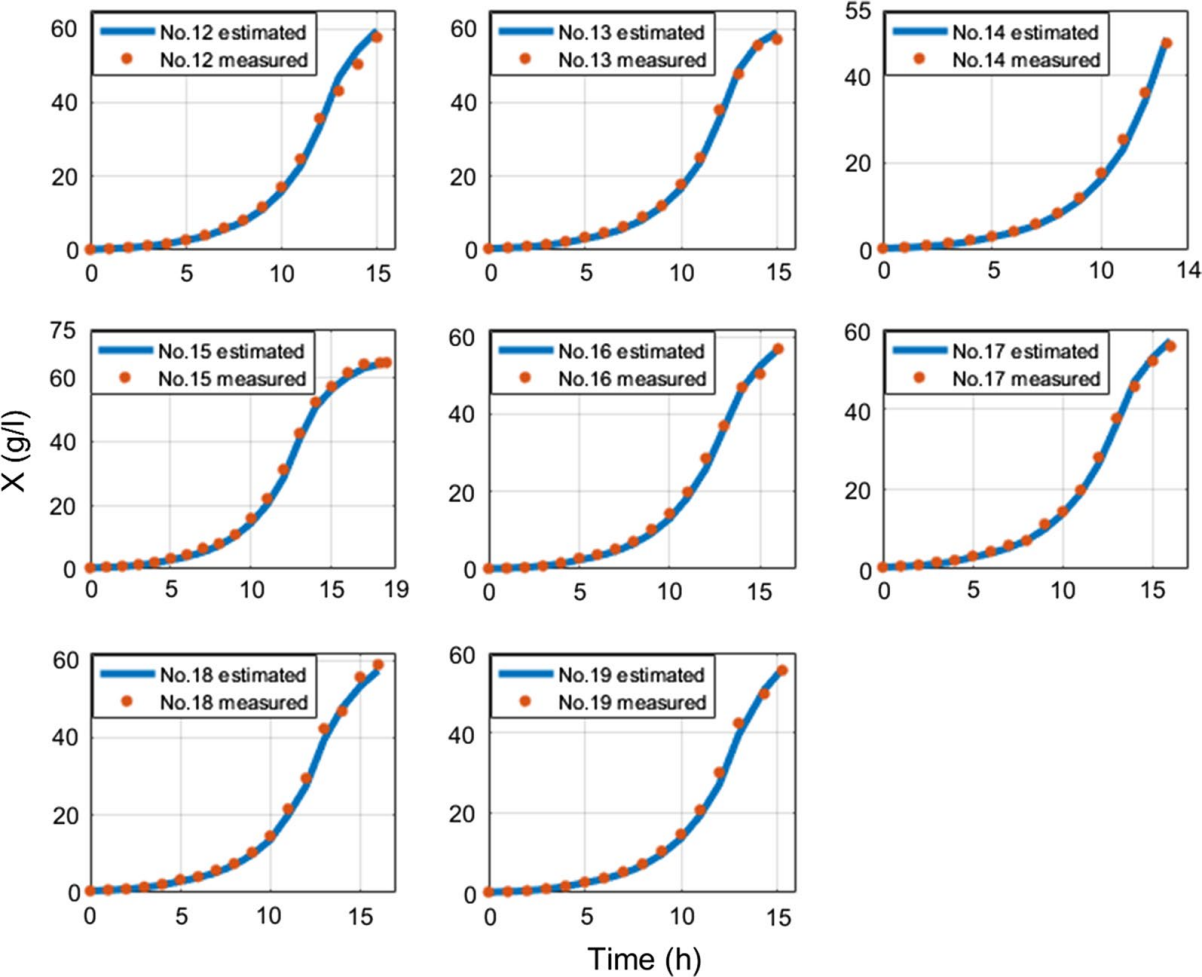
Biomass concentration estimation result with recombinant *E. coli* BL21 (DE3) pET21-IFN-alfa-5 strain at 7 L bioreactor with limited substrate feeding

**Fig. 12:**
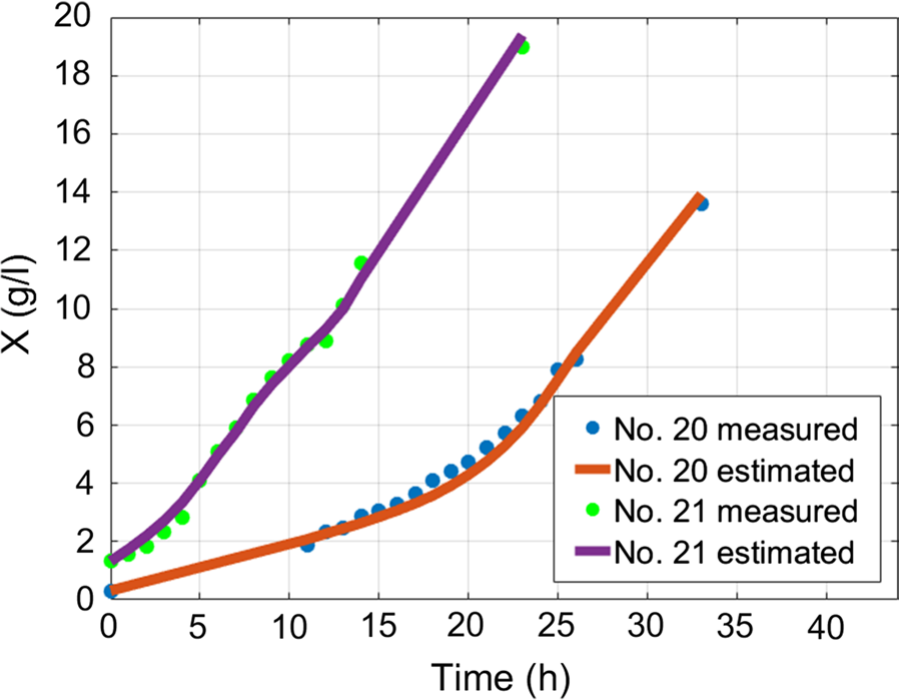
Biomass concentration estimation result with *Saccharomyces cerevisiae* (no DY7221) strain at 5 L bioreactor

The overall average MAE of biomass estimation since inoculation is 1.1 g/l and overall average MAE of biomass estimation since feed start is 1.41 g/l. The overall average MAPE of biomass estimation since inoculation is 7.28% and overall average MAPE of biomass estimation since feed start is 6.29%. Overall average RMSE value of *S. cerevisiae* cultivations is 0.5 g/l. RMSE value of *E. coli* cultivations with limited substrate feeding is 1.26 g/l and for cultivations with dosed substrate feeding is 2.44 g/l. RMSE value of *E. coli* cultivations before stationary phase, when DCW reaches ~ 40 g/l (to compare with results in [22]) with limited substrate feeding, is 1.07 g/l and for cultivations with dosed substrate feeding is 1.2 g/l. These results show that this approach improves the precision achieved in [22] without compromising the simplicity of the implementation. Offline analysis (stage A) execution lasted 2–15 ms and online analysis (stage B) calculations took 13–30 ms on a single core CPU in bioprocess engineering software tool dedicated for the purposes of this work. No initial conditions for numeric optimization procedure were used. The speed of online estimation can be explained by the fact that the prediction value of biomass concentration estimate is calculated once during the whole estimation procedure. There is no updating performed for the predicted value of biomass. In the future, this optimization condition might be released though. The substrate feed was started from the beginning of cultivation process right after inoculation moment in the experiments #1–#3 and #20–#21, while for the rest of cultivations had their substrate feed started after 5–6 h since inoculations. The errors between off-line and on-line data mainly originate from offline measurements. Especially in #5–#19, because historically the accuracy of offline measurements was not of high priority during these experiments. Therefore, in the future the true ground truth of biomass concentration might testify that the approach suggested in this work has even higher overall precision than the one stated in above. All biomass state estimation results are shown at the Figs. 8, 9, 10, 11, 12.

## Conclusions

The suggested biomass estimation’s numeric approach using cumulative oxygen uptake rate signal showed no dependability on selection of the initial variable values for optimization procedures. This study assumed, by Pareto principle, that the proposed method is only dependent on stoichiometry parameters of the strain, i.e. the developed noninvasive biomass estimation procedure was made to not depend on both the manipulation with a specific growth rate variable and the selection of corresponding bioreactor parameters. The precision errors, since the bioprocess start, when inoculant was injected to a bioreactor, confirmed that the approach is relevant for online biomass state estimation. This included the lag and exponential growth phases for both *E. coli* and *S. cerevisiae*. The experimental investigation of *E. coli* and *S. cerevisiae* cultures showed that the estimation procedure is identical for both cultures. The overall average MAE of biomass estimation since inoculation is 1.1 g/l and the overall average MAPE of biomass estimation since inoculation is 7.28%. RMSE value of *E. coli* cultivations before stationary phase, when DCW reaches ~ 40 g/l (to compare with results of other authors) with limited substrate feeding, is 1.07 g/l and for cultivations with dosed substrate feeding is 1.2 g/l. These results show that this approach improves the precision achieved by other authors without compromising the simplicity of the implementation. Moreover, the suggested approach is a candidate method to be the microorganisms’ culture invariant approach, it does not depend on any numeric initial optimization conditions, and it does not require any of bioreactor parameters. No numeric stability issues of convergence occurred during multiple performance tests. All this makes this approach a potential candidate for industrial tasks with adaptive feeding control or automatic inoculations when substrate feeding profile and bioreactor parameters are not provided.

Neither numeric artifacts nor abrupt worst-case scenarios were experienced during both offline and online analysis of 21 experiments, out of which 7 ones were carried out with dosed substrate feeding. The experiments executed in 5 l, 7 l, 12 l and 15 l bioreactor volumes. Feed start, inoculation, bioreactor medium, feeding limitation and other conditions varied with no manual control or adjustment. This encourages the use of such estimator in adaptive feedback control systems. Both online and offline estimations were tested on a single core CPU processing and each procedure took no more than 30 ms when overall 1-min interval data was sampled from cumulative oxygen uptake signal, which makes the approach of practical use too. Finally, this estimator does require a usage of regular industrial gas analysis equipment such as BlueSens etc.

## Data Availability

Some datasets used and analyzed during the current study are available from the corresponding author on reasonable request.
